# Functional analysis of four *LDLR* 5′UTR and promoter variants in patients with familial hypercholesterolaemia

**DOI:** 10.1038/ejhg.2014.199

**Published:** 2014-09-24

**Authors:** Amna Khamis, Jutta Palmen, Nick Lench, Alison Taylor, Ebele Badmus, Sarah Leigh, Steve E Humphries

**Affiliations:** 1 Centre for Cardiovascular Genetics, British Heart Foundation Laboratories, Institute Cardiovascular Science, University College London Medicine School, London, UK; 2 NE Thames Regional Genetics Service, Great Ormond Street Hospital, London, UK

## Abstract

Familial hypercholesterolaemia (FH) is an autosomal dominant inherited disease characterised by increased low-density lipoprotein cholesterol (LDL-C) levels. The functionality of four novel variants within the *LDLR* 5′UTR and promoter located at c.-13A>G, c.-101T>C, c.-121T>C and c.-215A>G was investigated using *in silico* and *in vitro* assays, and a systemic bioinformatics analysis of all 36 reported promoter variants are presented. Bioinformatic tools predicted that all four variants occurred in sites likely to bind transcription factors and that binding was altered by the variant allele. Luciferase assay was performed for all the variants. Compared with wild type, the c.-101T>C and c.-121T>C variants showed significantly lower mean (±SD) luciferase activity (64±8 and 72±8%, all *P*<0.001), suggesting that these variants are causal of the FH phenotype. No significant effect on gene expression was seen for the c.-13A>G or c.-215A>G variants (96±15 and 100±12%), suggesting these variants are not FH causing. Similar results were seen for the c.-101T>C and c.-121T>C variants in lipid-depleted serum. However, a significant reduction in luciferase activity was seen in the c.-215A>G variant in lipid-depleted serum. Electrophoretic-mobility shift assays identified allele-specific binding of liver (hepatoma) nuclear proteins to c.-121T>C and suggestive differential binding to c.-101T>C but no binding to c.-215A>G. These data highlight the importance of *in vitro* testing of reported *LDLR* promoter variants to establish their role in FH. The functional assays performed suggest that the c.-101T>C and c.-121T>C variants are pathogenic, whereas c.-13A>G variant is benign, and the status of c.-215A>G remains unclear.

## Introduction

**Figure 1:**
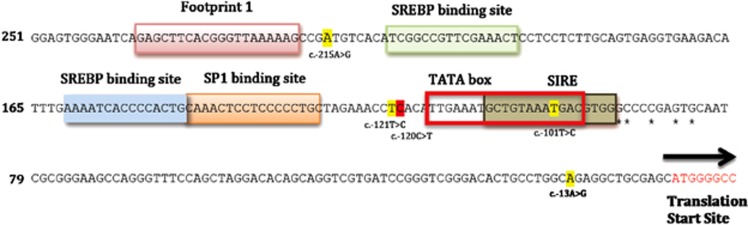
Illustration of the promoter and 5′UTR region of the *LDLR* gene. Highlighted boxes illustrate known binding sites. Variants examined in this study are highlighted in red, and highlighted in yellow is the positive control used. The start sites for transcription are indicated by *. The numbers to the left indicate the nucleotide number.

Familial hypercholesterolaemia (FH) is an autosomal dominant disease characterised by high levels of low-density lipoprotein cholesterol (LDL-C)^1^ that leads to an increased risk of premature coronary heart disease.^1^ Recent guidelines for the identification and management of patients with FH recommend that mutation information should be used to test all first-degree relatives to find carriers and offer them lifestyle and therapeutic interventions, such as lipid-lowering statin treatment, to reduce their risk of early heart disease.^2^ To date, variants in three genes have been implicated in FH, *low-density lipoprotein receptor* (*LDLR*), *apolipoprotein-B* (*APOB*) and *proprotein convertase subtilisin/kexin 9* (*PCSK9*).^3,4^ *LDLR* variants are the most common cause of FH, with >1200 variants identified throughout the gene (UCL LDLR variant database: https://grenada.lumc.nl/LOVD2/UCL-Heart/home.php?select_db=LDLR).^5,6^ At least 36 variants have been described in the 5′untranslated region (UTR) and promoter of the *LDLR* gene (Table 1), many of which have been shown to alter the binding of key transcription factors involved in the control of *LDLR* expression, such as Sterol regulatory element-binding protein (SREBP), specificity protein 1 (Sp1) and cAMP responsive element-binding protein (CREB).^7, 8, 9^ As not all identified variants will be causing FH, it is important that reported variants are tested for their pathogenicity. The functionality of four variants identified in the literature^6^ in patients with a diagnosis of heterozygous FH within the *LDLR* 5′UTR (c.-13A>G) or promoter (c.-101T>C, c.-121T>C and c.-215A>G) were analysed using *in silico* and *in vitro* assays. Previous *in vitro* luciferase results have shown that the variant c.-120C>T had a significant reduction in luciferase activity compared with the wild type.^10^ This variant was used as a positive control for these experiments. The location of known transcription factor binding sites in the *LDLR* promoter and the positions of the tested variants are shown in Figure 1.

## Materials and methods

### Subjects

The c.-101T>C and c.-215A>G variants were identified by the NE Thames Regional Genetics Service laboratories, Great Ormond Street Hospital (GOSH), in patients with a clinical diagnosis of heterozygous FH according to the UK criteria.^1^ The c.-13A>G and c.-121T>C variants were reported in FH patients in the literature.^11,12^ Standard nomenclature was used for describing variants (www.hgvs.org), and the locus reference genomic sequence LRG_274 for *LDLR* was used.

### *In silico* analysis

Two transcription factor prediction tools were used: MatInspector (www.genomatrix.de) and MATCH (http://www.gene-regulation.com/pub/programs.html#match).^13,14^ These tools were used to identify potential transcription factor binding sites in the region where the variants in the *LDLR* promoter were found. The matrix score calculates a match between the sequence and the matrix. The scores range from 0 to 1, with 1 indicating an exact match.

### Cloning of *LDLR* promoter

The c.-600 bp to c.-5 bp region of the *LDLR* promoter was cloned into the pGL2-basic vector using the *Hin*dIII restriction site.^8^ This was used as a template for site-directed mutagenesis using the QuikChange Lightning Site-Directed Mutagenesis Kit (210518-5; Agilent Stratagene Technologies, Stockport, UK). Site-directed mutagenesis was completed to create plasmids containing the c.-13G, c.-101C, c.-121C and c.-215G alleles. All constructs were confirmed by sequencing. The plasmids were transfected into Huh7 cells, a human hepatoma liver cell line (01042712; European collection of cell cultures (ECACC)). The cells were grown using Dulbecco's modified Eagle's medium (DMEM) with 10% FBS, or 10% lipid-depleted serum (from GENTAUR Europe BVBA, Aachen, Germany; FB-1090LF/100).

### Transfection and luciferase assay

Transfection was prepared when a confluence of 80% was reached. The pUC18 and pGL3 control were used as negative and positive controls, respectively. The cells were transfected using Lipofectamine 2000 (Invitrogen, Paisley, UK) and Opti-MEM serum-free medium according to the manufacturer's instructions and as previously described.^7^ Cells were lysed using Passive Lysis Buffer (Promega, Southampton, UK) and luciferase expression was determined using the Dual Luciferase Reporter Assay System (Promega), and measured in the Tropix TR717 Microplate Luminometer (PE Applied Biosystems, Paisley, UK). Luciferase activity was determined as the mean of 8–12 transfections with the assay performed in triplicate. EMSAs were carried out, as previously described, to determine transcription factor binding within the variant site.^8^

## Results

*In silico* analysis indicated that all of the variants were likely to be within transcription factor binding sites (Supplementary Table 1). With the exception of the predicted CREB site at c.-101T>C, none of these have been reported to be involved in control of transcription of the *LDLR* gene. The minor differences in predicted scores for the variants at the c.-121 site are unlikely to be of biological significance. For three of the variants (c.-13A>G, c.-101T>C and c.-215A>G), one or both of the programmes used predicted that binding was likely to be significantly modified by the presence of the variant allele, for example, the predicted binding of CREB to c.-101T but not c.-101C, and the binding of HIFIA to c.-101C and not c.-101T. However, although the c.-13G allele showed the abolition of two transcription factors, this binding was not verified in MATCH. Moreover, in MatInspector, the c.-120C>T variant showed an abolition of OCT1 binding site, indicating a biological significance. However, this result was not seen in the MATCH results.

**Figure 2:**
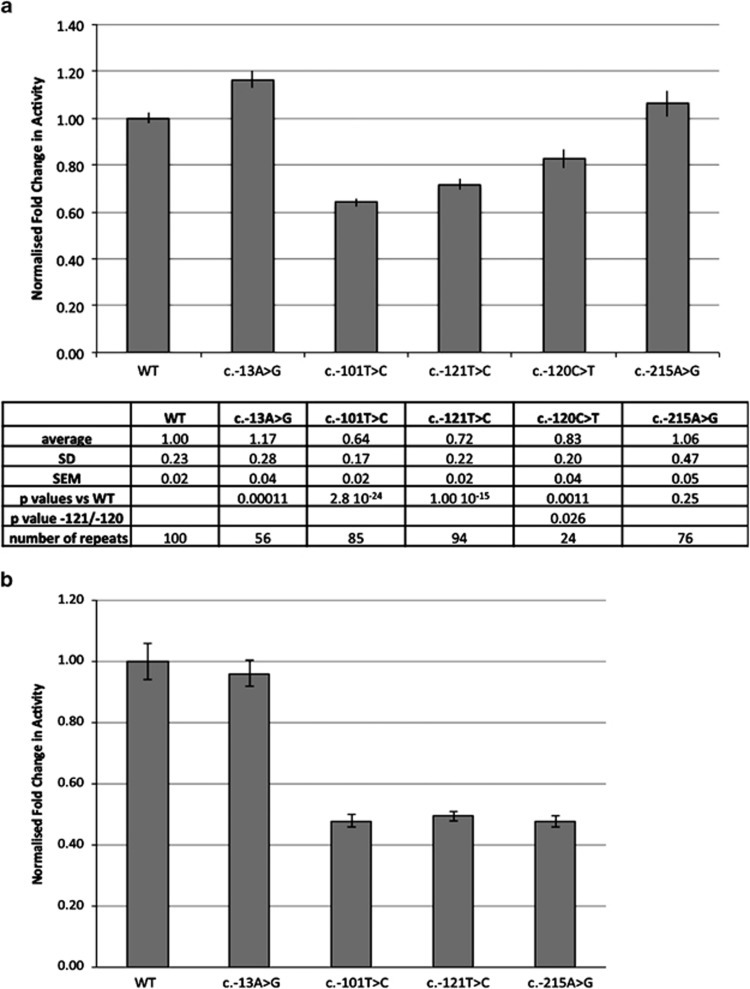
(**a**) Measurement of promoter activity using luciferase assay. Fragments containing the wild type and mutant were cloned into the pGL2 promoter, transfected into Huh7 cells and cultured using DMEM medium. Data were obtained in medium supplemented with 10% bovine calf serum. The mutants were compared with the wild type, normalised to the value of 1. These results are the average of 8–12 repeats in 3 independent experiments. Error bars indicate SEM. (**b**) Measurement of promoter activity using luciferase assay. Data were obtained in medium supplemented with 10% lipid-depleted bovine calf serum.

Luciferase assays were performed to investigate the *LDLR* expression in the promoter sequences containing the variant alleles compared with the wild type. These assays were repeated in multiples of 8–12 for each variant and are presented as the average of three independent runs. Compared with the wild type, the c.-13G and c.-215G alleles showed a (mean±SD) 96±15 and 100%±12% level of luciferase activity, indicating that this variant is unlikely to be FH causing. The c.-101T>C and c.-121T>C variants showed a 64±8 and 72±8% lower luciferase activity compared with the wild type (all *P*<0.001; Figure 2a), indicating that all these variants are likely to be FH causing. The c.-120C>T variant was then used as a positive control, showing 83% expression as compared with the wild type (*P*=0.001). All transfection assays were repeated using cells grown in 10% lipid-depleted serum in order to induce *LDLR* promoter expression.^8^ This resulted in a 2.5-fold higher expression of the wild-type construct, with similar increases seen for all variants (data not shown). However, as shown in Figure 2b, compared with the wild-type construct, the pattern of expression seen was similar, with the c.-13G allele showing a 96±22% level of luciferase activity, indicating that this variant is unlikely to be FH causing, whereas c.-101T>C and c.-121T>C variants showed 48±8 and 49±6%, respectively (all *P*<0.001). Remarkably, the c.-215G allele showed a similar and significant reduction in luciferase activity in the absence of lipid (48±10% *P*<0.001).

EMSAs were used in an attempt to identify nuclear factors binding differentially to the c.-101T>C, c.-121T>C and c.-215A>G variants. Extracts from a hepatocarcinoma cell line Huh7 were used, with a probe for the SREBP as a positive control. As shown in Supplementary Figure 1, the positive control provided a strong band shift as well as several nonspecific bands. Neither alleles of the c.-215A>G probe showed a specific additional band; however, the c.-121T allele showed an additional band shift compared with the c.-121C allele. For the c.-101 probes, both showed evidence of a specific high-molecular-weight band, with the c.-101C allele showing an additional faint band (indicated by an arrow in Supplementary Figure 1).

## Discussion

In order to correctly report to a referring clinician whether or not an identified promoter variant is likely to be FH causing, bioinformatics predictions may be helpful, and although evidence that the variant affects binding of a nuclear protein by an EMSA may be suggestive, an *in-vitro* assay of the effect on transcriptional strength provides the strongest evidence. Four reported variants identified in FH patients within the *LDLR* 5′UTR and promoter were investigated.^6^ The c.-13A>G variant showed no difference in luciferase activity, suggesting it is not FH causing, whereas c.-101C and c.-121C showed a significant lower expression, suggesting that these variants are likely to be pathogenic and causal of the FH phenotype seen in these patients, and the status of c.-215A>G remains unclear.

The bioinformatic programmes were not helpful in this instance. Both programmes (MatInspector and MATCH) suggested that the c.-13A>G variant destroys binding for a nuclear transcription factor and yet this variant did not affect luciferase activity. This is in agreement with previous studies that have shown no causal effect of variants within the 5′UTR region.^15^ Both programmes used also suggested that transcription factors would bind to the DNA sequences and that the variants would destroy binding. This is particularly clear for the c.-101T>C allele that is located in the TATA-box and a previously documented CREB binding site.^7^ Moreover, there were major differences between the two programmes; for example, although MatInspector showed a high score for the binding of two transcription factors, MATCH did not find any binding sites. Of the identified transcription factor binding sites (Supplementary Table 1), CREB is involved in *LDLR* expression. The SIRE (sterol-independent regulatory element), located c.-94 to c.-110 of the promoter, binds CREB in a cholesterol-independent manner.^7^ CREB was predicted to bind to the wild-type sequence, but was abolished in the variant sequence. Moreover, another predicted transcription factor, OCT1, has been implicated as being involved in *lipoprotein lipase* (*LPL*) expression, and is critical for hydrolysis of triglycerides in lipoproteins.^16,17^ Although OCT1 was predicted to bind by MatInspector and MATCH, there are no major differences in the scores for the variant and wild-type binding sites. These algorithms lack the complexity to take account of physiological conditions. The binding of transcription factors to a specific DNA sequence is dependent on various intracellular and extracellular stimuli within a cell. Therefore, although some transcription factors are predicted to bind to these sequences (with scores as high as 1.0), this does not take into account *in vivo* conditions such as chromatin structure, and are hence not ideal representations of what occurs in the cell. Bioinformatics tools should not be used in isolation but as a guide for experimental research.

The EMSA assay was also inconclusive: one out of the three functional sites determined by luciferase assay showed no evidence of binding of a nuclear protein, and this may be because of the protein requiring a longer binding site than the probe provided or nonphysiological binding conditions. Although we are not aware of any direct evidence for a role of HIF1A in the control of *LDLR* expression, this factor is involved in expression of *PCSK9*,^18^ a key gene involved in cholesterol metabolism, and hence the prediction of its binding here may be correct.

In order to put the present findings in context, we have carried out a systematic review of the published promoter and 5′UTR variants and examined the functional assay data reported. Including the four variants examined here, there are 36 variants reported, of which 20 have been directly tested using reporter assays, usually luciferase (Table 1). Of the four variants identified after the upstream SREBP1 binding site (which extends from c.-193 to c.-107), two have been tested and one (c.-217C>T) reported to have higher transcriptional activity and one (c.-208A>T) with no effect on luciferase activity, and thus neither are FH causing. The variant tested here at c.-215A>G has a modest effect on transcription and no EMSA binding was detected. This region contains sequences involved in the Footprint 1 element responsible for enhancement of *LDLR* transcription, with the key nucleotides identified at c.-218, c.-219 and c.-231,^19^ but no variants at these sites have been reported so far. Of the 23 variants between c.-207 and c.-90, where the binding sites for the key transcription factors SREBP1, SP1 and the TATA box CREBP are located, 14 have been tested and all found to have a profound effect on luciferase levels. Excluding the two examined here, the percentage of wild-type activity reported ranges from 0 to 40% (mean 15%). The modestly lower ~50% luciferase noted for the variants tested here (c.-215A>G, c.-121T>C and c.-101T>C) therefore raises the possibility that these may not be FH causing, although the data obtained are based on 8–12 replicates and three repeat experiments, and the lower transcriptional data are statistically robust. To address this, we have used c.-120C>T as a positive control that has been reported to have only 3% luciferase activity compared with the wild-type promoter sequence.^10^ In our hands, we observe that the variant has 83±9% activity compared with the wild type, confirming that the variant is FH causing but that the effect is not so drastic as previously observed. This could be because of the differences in cell culture conditions; for example, Francova *et al*^10^ used the HepG2 cell line compared with the Huh7 hepatoma cells that we used. However, the reduction in luciferase activity observed with the novel variants tested here are similar to the reported FH-causing c.-120C>T variant, strongly supportive of a causal effect of our variants. Finally, of the eight variants located after the start of transcription (c.-93 to c.-79), two have been tested and, as with the c.-13A>G tested here, none have a significant effect on promoter strength.^15^ Our data suggest that although there is no effect on luciferase activity for the c.-215G allele, there was a significant reduction in luciferase activity when supplemented in lipid-depleted conditions. In lipid-depleted conditions, SREBPs activate the transcription of *LDLR* by binding to SRE. Therefore, when lipid levels are low, *LDLR* expression is increased. However for the c.-215G allele construct, in lipid-depleted conditions, luciferase expression was reduced compared with the wild-type construct. Therefore, because lipid-depleted conditions mimic what occurs *in vivo*, these data support the view that this variant is possibly causal of the FH phenotype, but the status of this variant is still unclear, and co-segregation analysis in the relatives of this patient may resolve this issue. The data obtained from this study have been submitted to the UCL *LDLR* variant database (https://grenada.lumc.nl/LOVD2/UCL-Heart/home.php?select_db=LDLR).

There is high cross-species conservation across the whole of the *LDLR* promoter region and all the four sites tested here show strong conservation across six primate and six mammalian species (Table 1). It is noteworthy that the lowest sequence conservation is seen in sites in the 5′UTR, where the majority of the tested variants appear not to affect promoter strength, suggesting that this region does not have a critical role in control of gene expression. A limitation of the data reported here is that we do not have any relative samples available from these probands as the variants were identified from published reports,^11,12^ or from single samples sent to the diagnostic laboratory. Co-segregation of the variant with elevated lipid levels in relatives would strengthen the inference that they are FH causing. For all patients, DNA was tested for the common FH-causing mutations in the *APOB* and *PCSK9* genes and for the Portuguese patient the entire *PCSK9* gene was screened, but it remains a formal possibility that their elevated LDL-C levels may be caused by an unidentified variant in these genes. Finally, for the EMSAs we did not carry out detailed competition or supershift experiments to confirm the specificity or identity of the nuclear proteins binding to the alleles, as in our experience these type of experiments are difficult to quantify with accuracy and often give equivocal results that may not be of direct relevance to the *in vivo* situation because the examined element is not embedded within the full chromatin context.

*In vitro* luciferase reporter assays for identified promoter variants are important in determining the pathogenicity of promoter variants, allowing diagnostic reporting with greater confidence and subsequent cascade testing in the relatives of patients with these variations. This study highlights the difficulty of relying solely on *in silico* methods for the prediction of the pathogenicity of DNA sequence variants. Ideally, functional assays should be used to determine effects; however, the dilemma faced by many diagnostic service laboratories is that such tests are expensive and time consuming, and the relevant expertise and resources are rarely available in-house. Therefore, close links with research groups are key to the transfer of expert knowledge to diagnostic service laboratories. In conclusion, the functional assays performed indicate that c.-101T>C and c.-121T>C are likely to be pathogenic, whereas the c.-13A>G variant appears to be benign and the status of c.-215A>G remains unclear.

## Figures and Tables

**Table 1:** Location of all reported variants 270 bp upstream from LDLR start of translation, with reported effect on promoter activity and homology across human and 6 primate and 6 mammalian species (variants tested in this study are shown in bold)

*Variant*	*Promoter activity (% of wt)*	*Location*	*Sequence homology*^a^	*Reference*
c.-268G>T	In FH and normal	1 bp from FP1	7/8	^20^
c.-220_-221insA	Not tested	FP1	NA	^21^
c.-217C>T	160% Luciferase	2 bp from FP1	13/13	^20^
**c.-215A>G**	100% Luciferase	4 bp from FP1	11/12	^6^
c.-208A>T	100% Luciferase	Between SREBP1 and FP1	12/12	^15^
c.-206C>T	Not tested	Between SREBP1 and FP1	13/13	^22^
c.-193_-186delinsTG	Not tested	SREBP1	NA	^23^
c.-188C>T	Not tested	SREBP1	13/13	^23^
c.-185_-183delCTT	10% Luciferase	SREBP1	NA	^24^
c.-156C>T	Not tested^b^	SREBP2	13/13	^25^
c.-155_-154delinsTTCTGCAAACTCCT	11% Luciferase	SREBP2	NA	^15^
c.-153C>T	Not tested	SREBP2	13/13	^26^
c.-152C>T	40% Luciferase	SREBP2	13/13	^20^
c.-146C>A	Not tested	Between SREBP2 and SP1	13/13	^27^
c.-142C>T	20% Luciferase	SP1	13/13	^28^
c.-140C>G	7% Luciferase	SP1	13/13	^15^
c.-140C>T	6% Luciferase	SP1	13/13	^15^
c.-139C>A	Not tested	SP1	13/13	^23^
c.-139C>G	26% Luciferase	SP1	13/13	^8^
c.-138delT	24% LDLR activity	SP1	12/13	^29^
c.-138T>C	25% Luciferase	SP1	12/13	^30^
c.-137C>T	Not tested^c^	SP1	13/13	^31^
c.-136C>G	12% Luciferase	SP1	13/13	^15^
c.-136C>T	5% CAT assay	SP1	13/13	^32^
c.-135C>G	Not tested^d^	SP1	13/13	^31^
**c.-121T>C**	72% Luciferase	Between TATA box and SP1	13/13	^12^
c.-120C>T	3% Luciferase	Between TATA box and SP1	13/13	^26^
**c.-101T>C**	64% Luciferase	TATA BOX	12/12	^6^
c.-88G>A	100% Luciferase	5′UTR	12/12	^15^
c.-68A>C	Not tested	5′UTR	5/11	^33^
c.-36T>G	100% Luciferase	5′UTR	6/10	^15^
c.-23A>C	Not tested	5′UTR	7/12	^34^
c.-22delC	Not tested	5′UTR	10/12	^35^
c.-14C>A	Not tested	5′UTR	9/12	^27^
**c.-13A>G**	100% Luciferase	5′UTR	8/13	^11^
c.-5C>T	Not tested	5′UTR	7/11	^23^

Abbreviations: FP1, Foot Print 1; SREBP1, sterol regulating element-binding protein 1; SP1, Specificty Protein 1. Where the base for the sequence was not present in a particular species, the species was discounted from the total. The NG_009060.1 reference sequence was used.

a *LDLR* promoter sequence conservation between Human, *Pan troglodytes*, *Gorilla gorilla*, *Pongo abelii*, *Macaca mulatta*, *Callithrix jacchus*, *Oryctolagus cuniculus*, *Mus musculus*, *Rattus norvegicus*, *Bos taurus*, *Sus scrofa*, *Canis lupus familiaris* and *Equus caballus* (http://www.ensembl.org/Homo_sapiens/Gene/Compara_Alignments?align=609&db=core&g=ENSG00000130164&r=19%3A11200057-11244506&t=ENST00000252444).

b Cosegregation with FH reported.

c 5–15% LDLR activity (when compound heterozygote with c.1222G>A, p.(Glu408Lys)).

d 5–15% LDLR activity in homozygote.
