# Supplementary material for: Functional analysis of four LDLR 5′UTR and promoter variants in patients with familial hypercholesterolaemia
Source: Eur J Hum Genet. 2014 Sep 24;23(6):790–5. doi: 10.1038/ejhg.2014.199 (PMC4277481; doi:10.1038/ejhg.2014.199)
Supplement: Supplementary Information [file ejhg2014199x1.doc]

**Supplementary Figure 1:** Electro Mobility Shift Assay of the three *LDLR* functional promoter variants using an Huh7 nuclear extract

.
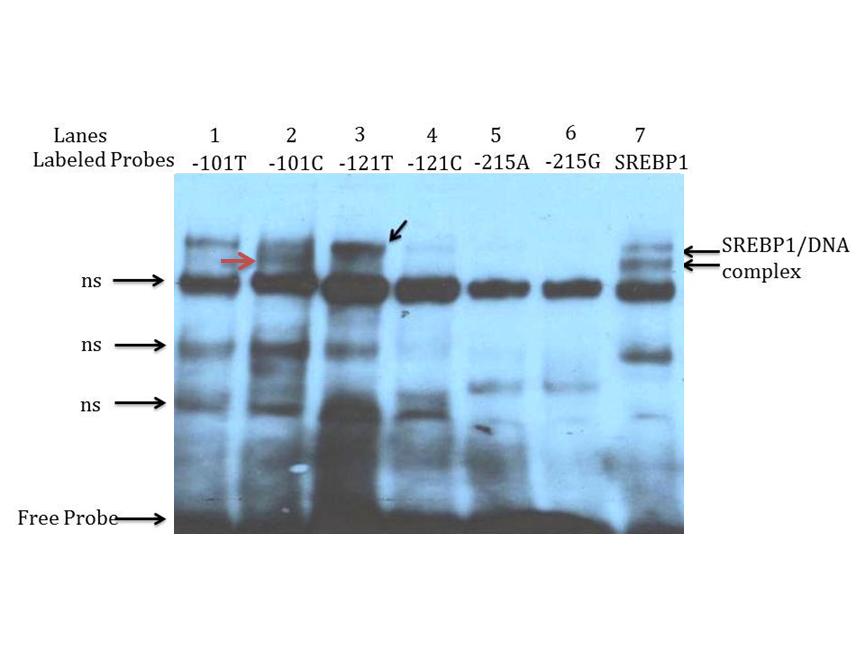


**Supplementary Figure 1:** **Electro Mobility Shift Assay of the three *LDLR* functional promoter variants using a Huh7 nuclear extract.**

Lanes 1-6 illustrate allele-specific DNA-protein interaction, following incubation of the nuclear extract with labelled probe for the wild type or variant probes for the three *LDLR* functional promoter variants. An allele-specific band was present in Lane 2 (-101C), indicated by an arrow. Lane 3 shows a specific binding for allele -121T, not present for -121C. Lanes 5 and 6 show display non-specific binding (ns), present in all probes examined and unaffected by the variant. As a positive control to demonstrate successful binding, Lane 7 shows binding of nuclear extract to the SREBP-1 consensus sequence probe, with the resulting SREPB-1-specific bands indicated by arrows. The red arrow indicates the additional faint band detected in the -101C allele.

Supplementary Table 1: Bioinformatic Analysis of Studied Variants

| Nucleotide change | TF | MatInspector | | MATCH | | Relevance |
| --- | --- | --- | --- | --- | --- | --- |
|  |  | WT | Mutant | WT | Mutant |  |
| c.-13A>G | PAX5 | 0.861 | -- | -- | -- |  |
|  | NF1 | 0.975 | -- | -- | -- |  |
| c.-101T>C | TEF | 0.854 | 0.854 | -- | -- |  |
|  | TCF11 | 1.000 | --- | -- | -- |  |
|  | HIF1A | -- | 0.976 |  | -- |  |
|  | CREB | 0.905 | -- | 0.926 | -- | Binds to *LDLR* promoter^1^ |
|  | ATF6 | 0.977 | --- | 0.971 | -- |  |
|  | XBP1 | -- | 0.900 | 0.910 | -- |  |
|  | JUN | -- | -- | 0.971 |  |  |
| c.-121T>C | BCL6 | 0.821 | 0.835 | -- | -- |  |
|  | AML3 | 0.865 | 0.855 | -- | -- |  |
|  | EN1 | 0.781 | 0.781 | -- | -- |  |
|  | OCT1 | 0.902 | 0.902 | 0.970 | 0.970 | *LPL* expression^2^ |
| c.-120C>T | BCL6 | 0.835 | 0.835 | -- | -- |  |
|  | OCT1 | 0.902 | -- | 0.970 | 0.964 |  |
|  | SOX/SRY | -- | 0.847 | -- | -- |  |
| c.-215A>G | BARB (BOX) | 0.881 | 0.894 | 0.902 | 0.911 |  |
|  | E4F1 | 0.825 | --- | -- | -- |  |
|  | SIX4 | --- | 0.960 | -- | -- |  |

TF: transcription factor;;WT: Wild type; ---: No binding predicted

^1^ Reported to bind to *PCSK9* promoter , ^2^ Reported to bind to *LDLR* promoter

**Supplementary Material**

Probes used for the EMSA study

-101TF 5’TGAAATGCTGTAAATGACGTGGGCCCCGA3’

-101TR 5’TCGGGGCCCACGTCATTTACAGCATTTCA3’

-101CF 5’TGAAATGCTGTAAACGACGTGGGCCCCGA3’

-101CR 5’TCGGGGCCCACGTCGTTTACAGCATTTCA3’

-121TF 5’CCCTGCTAGAAACCTCACATTGAAATGCT3’

-121TR 5’AGCATTTCAATGTGAGGTTTCTAGCAGGG3’

-121CF 5’CCCTGCTAGAAACCCCACATTGAAATGCT3’

-121CR 5’AGCATTTCAATGTGGGGTTTCTAGCAGGG3’

-215GF 5’GGGTTAAAAAGCCGATGTCACATCGGCCG3’

-215GR 5’CGGCCGATGTGACAACGGCTTTTTAACCC3’

-215GF 5’GGGTTAAAAAGCCGGTGTCACATCGGCCG3’

-215GR 5’CGGCCGATGTGACACCGGCTTTTTAACCC3’

References:

1. Li H, Dong B, Park SW, Lee HS, Chen W, Liu J. Hepatocyte nuclear factor 1alpha plays a critical role in PCSK9 gene transcription and regulation by the natural hypocholesterolemic compound berberine. J. Biol. Chem. 2009;284(42):28885-95. doi:10.1074/jbc.M109.052407

2. Liu J, Ahlborn TE, Briggs MR, Kraemer FB. Identification of a novel sterol-independent regulatory element in the human low density lipoprotein receptor promoter. J. Biol. Chem. 2000;275(7):5214-21.
